# Efficacy and safety of Shenfu injection in the treatment of sepsis: A protocol for systematic review and meta-analysis

**DOI:** 10.1097/MD.0000000000027196

**Published:** 2021-09-17

**Authors:** Shu Luo, Lianping Gou, Shiping Liu, Xiaoping Cao

**Affiliations:** a Emergency Department, Affiliated Hospital of North Sichuan Medical College, Nanchong, Sichuan Province, China; b General Medical Department, Affiliated Hospital of North Sichuan Medical College, Nanchong, Sichuan Province, China.

**Keywords:** meta-analysis, protocol, sepsis, Shenfu injection

## Abstract

**Background:**

Sepsis is a syndrome of infection-induced systemic inflammatory response. Conventional treatment combined with Shenfu injection (SFI) has been previously validated clinical effective in alleviating inflammatory response in patients with septic shock. However, evidence-based medical evidence is scant. Herein, we designed the protocol of a proposed study based on the Preferred Reporting Items for Systematic Reviews and Meta-analyses guidelines, aiming to systematically evaluate the efficacy and safety of SFI in patients with sepsis.

**Methods:**

Eligible studies reporting the efficacy and safety of SFI in the treatment of sepsis published before August 2021 will be searched from online databases, including the PubMed, Web of Science, EMBASE, Ovid, the Cochrane Library, Wanfang Database, China National Knowledge Infrastructure, and China Biology Medicine Disc. The literature selection process will be reported in accordance with the Preferred Reporting Items for Systematic Reviews and Meta-analysis guidelines. After data extraction and methodological quality evaluation, Stata 12.0 software will be used to synthesize the data through fixed/random effects of meta-analysis models.

**Results:**

The results of this meta-analysis will be submitted to a peer-reviewed journal for publication.

**Conclusion:**

This study will provide reliable evidence-based basis for the clinical application of SFI in the treatment of sepsis.

**OSF Registration number:**

DOI 10.17605/OSF.IO/KCMDQ.

## 1 Introduction

Sepsis is a systemic inflammatory response caused by infection, which is a common critical illness.^[1,2]^ It is estimated there are more than 1.8 million patients with severe sepsis worldwide each year.^[3]^ Sepsis is featured by the rapid progression and has high mortality, which can be as high as 80% in sepsis complicated with infectious shock. As a result, sepsis is one of the major causes of death in critically ill patients.^[3]^ Therefore, the treatment of sepsis has been a hot spot in clinical research.

The development of sepsis is closely related to the release of inflammatory mediators from the human bodies, the spread or translocation of bacterial endotoxins, and coagulation disorders.^[4,5]^ Sepsis can further develop into septic shock (infectious shock).^[6]^ Septic shock is a syndrome of sepsis with severe cellular, metabolic and circulatory disturbances, and its risk of death is significantly higher than that of sepsis alone.^[7]^ As a major event during the disease process, the inflammatory response produces a cascade of waterfall reactions alongside the release of abundant inflammatory factors, and organ dysfunction occurs in severe cases.^[8]^ It is suggested that the inflammatory response is an important factor for the occurrence of septic shock.^[9]^ Currently, the conventional treatments of septic shock, including fluid resuscitation, anti-infection, anticoagulation, administration of vasoactive drugs and glucocorticoids, are important to stabilize blood pressure and reduce the inflammatory response.^[10–12]^ However, they are supportive managements that cannot target the individualized cause of sepsis. About 1/3 of patients with sepsis do not have a clear clue of pathogenic bacteria.^[13]^ Therefore, it is urgent to propose new treatment options for sepsis.

Traditional Chinese medicine (TCM) believes that sepsis belongs to the category of heat illness in typhoid and warm diseases, and it may also be related to Wei, Qi, Ying and Blood.^[14]^ The basic pathogenesis of septicemia is the imbalance of the internal organs due to the deficiency of the root and the prevalence of evil.^[15]^ Therefore, the TCM theory highlights the support of root cause in the treatment of sepsis. Shenfu injection (SFI) is derived from the ancient formula Shenfu decoction,^[16]^ which mainly contains the TCM herbals ginseng and radix aconiti carmichaeli. Ginseng has the effect of strengthening the vital energy, restoring the pulse and fixing the detachment, tonifying the spleen and benefiting the lung, generating fluid and quenching thirst, and calming the mind and educating the mind. Radix aconiti carmichaeli has the effect of returning Yang to rescue the rebellion, tonifying heat and helping Yang, dispersing cold and relieving pain.^[17]^ Through benefiting Qi and returning Yang, SFI strengthens the positive energy and enhances organ functions.

SFI is mainly composed of ginsenosides, aconitine, and other active ingredients, which have strong effects on maintaining blood pressure, stabilizing heart rate, and reducing pathological damage.^[18–20]^ It is confirmed that SFI combined with glucocorticoids can improve the immune function and reduce the level of inflammatory factors in patients with severe sepsis.^[21]^ Numerous studies have validated the function of SFI in improving the prognosis of sepsis, but controversy remains and evidence-based medical evidence is scant.^[21–26]^ This study aims to provide evidence-based medical evidence for the efficacy and safety of SFI in the treatment of sepsis through a systematic review and meta-analysis.

## 2 Methods

### 2.1 Protocol registration

This protocol was registered on the open science framework (Registration Number: DOI 10.17605/OSF.IO/KCMDQ). We will strictly perform this protocol by following the Preferred Reporting Items for Systematic Reviews and Meta-analyses Protocols statement guidelines.^[27]^

### 2.2 Inclusion criteria for study selection

#### 2.2.1 Type of studies

Only randomized controlled trials about SFI for the treatment of sepsis will be included published language will be limited in English or Chinese, and blinding method will not have a restriction.

#### 2.2.2 Type of participants

All patients with sepsis will be included without limitation of the age, race, and disease severity.

#### 2.2.3 Type of interventions

##### 2.2.3.1 Experimental interventions

Experimental interventions will include conventional treatment combined with intravenous administration, intravenous drip or intramuscular administration of SFI. The dose and frequency of SFI administration will be unlimited.

##### 2.2.3.2 Control interventions

Control interventions will involve conventional treatments like fluid resuscitation, anti-infection, anticoagulation, vasoactive drugs, and glucocorticoids.

#### 2.2.4 Type of outcome measures

##### 2.2.4.1 Primary outcomes

The 28-day mortality.

##### 2.2.4.2 Additional outcomes

1. Total effective rate;
2. Length of ICU stay;
3. Acute physiology and chronic health evaluation II;
4. Inflammatory indicators, including tumor necrosis factor-α (TNFα), serum procalcitonin (PCT), C-reactive protein (CRP), interleukin-1 (IL-1), interleukin-6 (IL-6), interleukin-10 (IL-10), etc; and
5. Adverse events.

### 2.3 Exclusion criteria

1. Retrospective studies, and
2. Studies with incomplete important information.

### 2.4 Searching strategy

A systematic searching of relevant literatures reporting the efficacy and safety of SFI in the treatment of sepsis published before August 2021 in PubMed, Web of Science, EMBASE, Ovid, the Cochrane Library, Wanfang Database, China National Knowledge Infrastructure, and China Biology Medicine Disc will be performed. The searching strategies in the PubMed were shown in Table 1, which will be used in other online databases. Literatures will be limited in Chinese and English language, without restriction on publication status.

**Table 1:** PubMed search strategy.

Number	Search terms
#1	Sepsis[MeSH]
#2	Pyaemia[Title/Abstract]
#3	Pyemia[Title/Abstract]
#4	Pyohemia[Title/Abstract]
#5	Blood poisoning[Title/Abstract]
#6	Poisoning, blood[Title/Abstract]
#7	Septicemia[Title/Abstract]
#8	Severe sepsis[Title/Abstract]
#9	Blood poisonings[Title/Abstract]
#10	Poisonings, blood[Title/Abstract]
#11	Pyaemias[Title/Abstract]
#12	Pyemias[Title/Abstract]
#13	Pyohemias[Title/Abstract]
#14	Sepsis, severe[Title/Abstract]
#15	Septicemias[Title/Abstract]
#16	OR/1 to 15
#17	Shenfu injection[Title/Abstract]
#18	Randomized controlled trials as topic[MeSH]
#19	Clinical trials, randomized[Title/Abstract]
#20	Controlled clinical trials, randomized[Title/Abstract]
#21	Trials, randomized clinical[Title/Abstract]
#22	Random∗[Title/Abstract]
#23	OR/18 to 22
#24	#16 and #17 and #23

### 2.5 Study selection and data extraction

#### 2.5.1 Selection of studies

**Figure 1:**
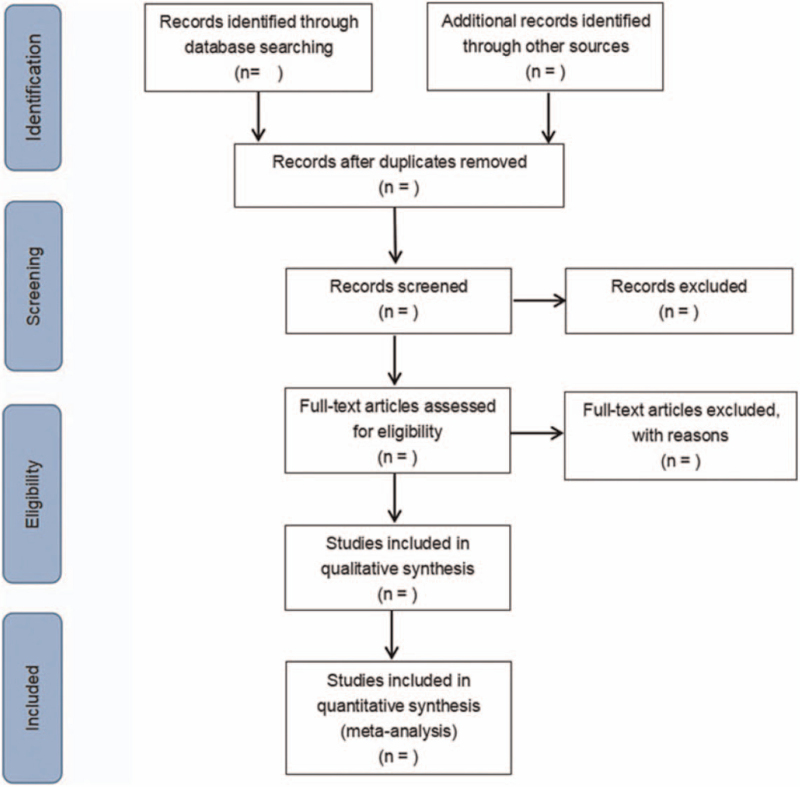
Flow diagram of literature retrieval.

A Preferred Reporting Items for Systematic Reviews and Meta-analysis flow chart will be drawn to illustrate the study selection procedure (Fig. 1). According to the research objectives and inclusion criteria, 2 researchers will independently read the literatures and extract data. Any disagreement will be solved by discussing with the third researcher.

#### 2.5.2 Data extraction and management

The following data will be extracted:

1) Literature source and publication date of the study title,
2) Age of subjects in experimental group and the control group, intervention measures and the implementation of the experiment,
3) Study type and relevant elements of bias risk assessment, and
4) Outcome indicators and outcomes.

#### 2.5.3 Risk of bias in the included studies

Two researchers will independently evaluate the risk of bias for each study using the Cochrane Collaboration tool, including random sequence generation, allocation concealment, blinding, incomplete outcome data, selective outcome reporting, and other potential sources of bias. Each domain will be ranked as low risk of bias, high risk of bias or unclear risk of bias.

#### 2.5.4 Measures of treatment effects

For dichotomous outcomes, risk ratio will be used in the meta-analysis. For continuous variables, the standardized mean difference with 95% confidence intervals will be selected. All of these data will be analyzed with the corresponding 95% confidence intervals.

#### 2.5.5 Dealing with missing data

Insufficient or missing data in the literature will be required by e-mailing the authors. If not available, only the current available data will be analyzed and the potential impacts will be discussed.

#### 2.5.6 Assessment of heterogeneity

Heterogeneity will be tested by Q-statistic and I^2^-statistic. I^2^ > 50% will be considered as significant heterogeneity, and the random-effects model or the fixed-effects model will be adopted.

#### 2.5.7 Data synthesis and meta-analysis

Stata 12.0 software (STATA Corporation, College Station, TX) will be used to combine and calculate the outcomes. If the heterogeneity is not significant (*P* > .1 or I^2^ < 50%), the fixed effect model will be adopted; Otherwise (*P* < .1 or I^2^ ≥ 50%), the random effect model will be chosen. Two-tailed *P* < .05 indicates statistical significances.

#### 2.5.8 Subgroup analysis

Subgroup analyses based on the dose of SFI, treatment durations and different types of routes of administration will be performed.

#### 2.5.9 Sensitivity analysis

Sensitivity analyses will be performed by removing the studies with high risk of bias or missing data.

#### 2.5.10 Assessment of publication bias

Publication bias will be assessed by depicting funnel plots if a sufficient number of trials (more than 10) are included.^[28]^

#### 2.5.11 Ethics and dissemination

Since the program does not include the recruitment of patients and the collection of personal information, it does not require the approval of the Ethics Committee.

## 3 Discussion

Sepsis is a common critical clinical condition with a high mortality. Although anti-inflammatory drugs and immune-enhancing agents like glucocorticoids and thymidine alpha 1 have been widely used in clinical practice, their adverse effects are becoming increasingly prominent.^[29]^ In recent years, with the continuous development of clinical research, TCM treatment of sepsis has been widely recognized. Among them, SFI has been frequently used in the treatment of sepsis because of its effect on returning Yang and rescuing the rebellion, and supporting the righteousness and fixing the detoxification.^[30]^ Although the therapeutic effectiveness of SFI for sepsis has been reported, evidence-based medical evidence is lacked. This meta-analysis will provide a detailed summary and analysis of the most recent evidence. We hope that our findings will help patients, clinicians, and healthcare policy makers to develop optimal TCM treatment options for patients with sepsis.

## Author contributions

**Conceptualization:** Xiaoping Cao, Shu Luo.

**Data curation:** Shu Luo, Lianping Gou.

**Formal analysis:** Xiaoping Cao, Shu Luo.

**Investigation:** Lianping Gou.

**Methodology:** Lianping Gou.

**Resources:** Lianping Gou.

**Software:** Shiping Liu.

**Supervision:** Xiaoping Cao.

**Validation:** Shiping Liu.

**Visualization:** Shiping Liu.

**Writing – original draft:** Xiaoping Cao, Shu Luo.

**Writing – review & editing:** Xiaoping Cao, Shu Luo.
